# Alterations of Circulating Bone Marrow–Derived VEGFR-2^+^ Progenitor Cells in Isolated Limb Perfusion With or Without rhTNF-α

**DOI:** 10.1245/s10434-012-2637-3

**Published:** 2012-09-05

**Authors:** Kai Nowak, Nicole Jachol, Neysan Rafat, Elena Joas, Grietje Ch. Beck, Peter Hohenberger

**Affiliations:** 1 Division of Surgical Oncology and Thoracic Surgery, Department of Surgery, Mannheim University Medical Center, Heidelberg University, Heidelberg, Germany; 2 Department of Anaesthesiology and Critical Care Medicine, Mannheim University Medical Center, Heidelberg University, Heidelberg, Germany; 3 Department of Pediatrics I, University Children’s Hospital Heidelberg, University of Heidelberg, Heidelberg, Germany

## Abstract

**Background:**

Circulating endothelial progenitor cells (cEPCs) as recruited to the angiogenic vascular system of malignant tumors have been proposed as a biomarker in malignancies. The effect of antitumor chemotherapy on cEPCs is not fully understood. We examined the level of cEPCs, vascular endothelial growth factor (VEGF), and angiopoietin-2 in the blood of sarcoma and melanoma patients before and after isolated limb perfusion (ILP) with or without recombinant human tumor necrosis factor-α (rhTNF-α).

**Methods:**

Twenty-two patients, 11 each with soft tissue sarcoma or recurrent melanoma of the limb, were recruited. ILP was performed with rhTNF-α/melphalan (TNF) or melphalan only (no TNF). Fifteen healthy volunteers served as control subjects. Blood was sampled before and up to 6 weeks after ILP. Peripheral blood mononuclear cells were isolated by density gradient centrifugation, and annexin V-negative cells were characterized as cEPCs by triple staining for CD133^+^, CD34, and VEGFR-2^+^.

**Results:**

Before treatment, cEPC numbers were significantly increased in sarcoma (0.179 ± 0.190 %) and melanoma patients (0.110 ± 0.073 %) versus healthy controls (0.025 ± 0.018 %; *P* < 0.01), but did not differ significantly between sarcoma and melanoma patients. cEPC decreased significantly after ILP in patients with no TNF compared to pretreatment values (*P* < 0.05) and were significantly lower at 4 h, 48 h, and 1 week compared to ILP with TNF (*P* < 0.05). Values 6 weeks after ILP were significantly lower than before ILP in both investigated groups (*P* < 0.01).

**Conclusions:**

ILP with TNF results in activation of bone marrow–derived EPCs compared to ILP without TNF. Alteration of cEPCs and angiopoietin-2 by rhTNF-α might account for the cytotoxicity and hemorrhagic effects on tumor vessels during limb perfusion procedures.

**Electronic supplementary material:**

The online version of this article (doi:10.1245/s10434-012-2637-3) contains supplementary material, which is available to authorized users.

Isolated limb perfusion (ILP) with recombinant human tumor necrosis factor-α (rhTNF-α, or tasonermin) and melphalan is a highly effective treatment for soft tissue sarcoma and in-transit metastases of malignant melanoma of the extremities. In a neoadjuvant setting, ILP contributes to radical resection of locally advanced sarcomas and long-term limb salvage.^1^–^3^

One of the major systemic side effects of rhTNF-α treatment is the induction of a systemic inflammatory response syndrome (SIRS), in which patients may develop tachycardia and fever as a result of increased cardiac output and decreased vascular resistance.^3^–^5^ The incidence and severity of SIRS correlates with the subsequent activation of the cytokine network and the occurrence of leakage from the limb to the systemic circulation.^2^ Regionally, the application of rhTNF-α yields an endothelial damage of tumor vessels with destruction of microcirculation and consecutive development of tumor necrosis.^6^,^7^ To our knowledge, the effect of rhTNF-α on bone marrow–derived (BMD) endothelial progenitor cells has not yet been described.

Tumor vascularization is dependent on the sprouting of nearby blood vessels, with migration and differentiation of existing mature endothelial cells (angiogenesis). Several studies have provided evidence that tumor vasculature can also arise through vasculogenesis, a process by which BMD endothelial progenitor cells (EPC) are recruited and differentiate into mature endothelial cells to form new blood vessels.^8^,^9^ Hematopoietic (VEGFR-1^+^) and endothelial (VEGFR-2^+^) BMD progenitors collaborate in disease progression, first by initiating the premetastatic niche and second by promoting the vascularization of metastatic lesions.^10^–^12^

Although a close interplay between EPCs and tumor neovascularization is suggested, the exact role of EPCs to the pathogenesis of undifferentiated tumors with high proliferation rates remains to be determined.^13^ Monitoring and targeting BMD endothelial progenitors is of interest to guide the optimal use of target therapies in patients.^13^–^15^ In this regard, the effect of rhTNF-α on BMD endothelial progenitor cells has not yet been studied.

We investigated the effect of a local administered drug combination targeting the tumor vasculature for antitumor treatment of ILP with rhTNF-α versus ILP with chemotherapy alone on cEPC in melanoma and high-grade sarcoma patients. Beside clinical parameters, the cEPC mobilizing factors vascular endothelial growth factor (VEGF) and angiopoietin were determined in blood to gather further information. We also assessed associated pathophysiologic changes affecting cEPC subordinate to applied drugs for antitumor treatment.

## Methods

### Patients

Eleven patients with high-grade soft tissue sarcoma (4 men, 7 women) and 11 patients with in-transit metastasized melanoma (3 men, 8 women) were enrolled onto this study. Patient characteristics are listed in Table 1. ILP with rhTNF-α/melphalan was used for in-transit metastasized melanoma (*n* = 5) and G3 sarcoma (*n* = 11) to be compared to ILP after treatment with cisplatin/melphalan alone for in-transit metastasized melanoma (*n* = 6). Exclusion criteria for the study were cardiogenic, septic, or hemorrhagic shock within the last 6 months; and pneumonia or a history of acute respiratory distress syndrome, pleural empyema, or any other sign of infection. None of the patients received concurrent medication known to influence the mobilization of cells from the bone marrow (i.e., hydrocortisone, ACE inhibitors, statins) at the time of blood sampling. For controls, we recruited 15 healthy volunteers from our laboratory staff. Informed consent was obtained from all study participants. The study was approved by the ethics committee of the University of Heidelberg.

**Table 1:** Patients treated with ILP with or without TNF

ILP with:	Disease	Sex	Age (y)^a^	Tumor type	Location
TNF (2 mg)	Sarcoma (*n* = 11)	F	65	Malignant fibrous histiocytoma G3	Left lower limb
Melphalan (10 mg/L perfused limb volume)		F	56	Malignant fibrous histiocytoma G3	Left elbow
		M	54	Dedifferentiated liposarcoma G3	Left thigh and knee
		F	40	Undifferentiated sarcoma G3	Right thigh and knee
		M	22	Epithelioid sarcoma G3	Left fore foot
		M	72	Myxofibrosarcoma G3	Left lower limb
		F	37	Synovial sarcoma	Right knee
		F	71	Myxoid liposarcoma G3	Left lower limb
		M	67	Epithelioid sarcoma G3	Right forearm
		F	34	Myxoid liposarcoma G3	Right thigh
		F	34	Liposarcoma G3	Left forearm
	Melanoma (*n* = 5)	F	59	In-transit metastasizing MM	Right lower limb
		M	85	In transit metastasizing MM	Left lower limb
		M	51	In-transit metastasizing MM	Right lower limb
		F	75	In-transit metastasizing MM	Left lower limb
		F	73	In-transit metastasizing MM	Right knee
Melphalan (10 mg/L perfused limb volume)	In-transit metastasized melanoma (*n* = 6)	F	69	In-transit metastasizing MM	Right lower limb
		M	36	In-transit metastasizing MM	Right lower limb
		F	75	In-transit metastasizing MM	Left lower limb
		F	45	In-transit metastasizing MM	Left lower limb
		F	75	In-transit metastasizing MM	Left lower limb
		F	51	In-transit metastasizing MM	Left lower limb

*ILP* isolated limb perfusion, *TNF* recombinant human tumor necrosis factor-α, *MM* malignant melanoma

^a^No significant differences concerning age were observed between the treatment groups

### Isolation Perfusion and Application of Drugs

The perfused limb volume was measured with the water displacement method.^16^ The detailed method of ILP has been described previously.^17^ Shortly after exposition and cannulation of the major artery and vein of the limb, extracorporeal circulation was established with a roller pump and heat exchanger (Jostra HL 20, Maquet, Germany). Gas exchange was achieved with a bubble oxygenator (Baxter, Utrecht, The Netherlands). The perfusate temperature ranged from 39 °C to 43 °C, and the volume of the perfusate was kept constant at approximately 700 ml. Tissue temperature was measured by needle probes inserted to healthy muscle and tumor tissue and was intended to be ≥38 °C and kept <40.5 °C. Perfusion time was 90 min. Leakage control was performed by injection of indium-111-labeled autologous erythrocytes and ^99m^Tc-labeled albumin to the limb circuit and continuous monitoring of the systemic circulation. After perfusion, the limb was rinsed with 3 L of hydroxyethyl starch until no further reduction of the radiopharmaceutical activity in the limb was achievable. rhTNF-α (Boehringer Ingelheim, Ingelheim, Germany) was provided at a dose of 2 mg (upper limb) or 3 mg (lower limb). The melphalan dosage was 10 mg/L of perfused limb volume, as described previously.

### Blood Sampling

In patients and healthy controls, 25 ml of blood was obtained by insertion of a 20-gauge cannula intravenously and collected in tubes containing sodium citrate (0.105 M) as an anticoagulant. Blood samples from patients were collected before ILP and 2 h, 4 h, 24 h, 48 h, 1 week, and 6 weeks after ILP.

### Flow Cytometry

All blood samples were processed within 1 h after collection. Peripheral blood mononuclear cells (PBMCs) were prepared by density gradient centrifugation with Ficoll-Hypaque (Amersham Biosciences, Freiburg, Germany). The expression of cell-surface antigens was determined by four-color immunofluorescence staining as described previously.^14^,^18^ Briefly, 100 μl of PBMC (containing 1 × 10^6^ cells) were incubated with 10 μl of FcReceptor-blocking reagent (Miltenyi Biotec, Bergisch-Gladbach, Germany) for 10 min to inhibit nonspecific bindings. The cells were then incubated at 4 °C for 30 min with 10 μl phycoerythrin (PE)-conjugated anti-human CD133 monoclonal antibodies (mAb) (Miltenyi Biotec, Bergisch-Gladbach, Germany), 10 μl Peridinin Chlorophyll Protein Complex (PerCP)-conjugated anti-human CD34 mAb (BD Biosciences, Heidelberg, Germany), 10 μl allophycocyanin (APC)-conjugated vascular endothelial growth factor receptor (VEGFR)-2 mAb (R&D Systems, Wiesbaden-Nordenstadt, Germany) and 10 μl fluorescein isothiocyanate (FITC)-conjugated annexin V mAb (BD Biosciences, Heidelberg, Germany). PE-, PerCP-, APC-, and FITC-conjugated isotype-matched immunoglobulin (Ig)-G1 and IgG2a antibodies (DakoCytomation, Hamburg Germany) were used for each patient and measurement as negative controls. The cells were washed three times to remove unbound antibodies and finally resuspended in 400 μl of fluorescence-activated cell sorting (FACS) solution (BD Biosciences, Heidelberg, Germany). FACS analysis was performed on a FACSCalibur flow cytometer (BD Biosciences, Heidelberg, Germany) and the data were analyzed by WinMDI 2·8 software (developed by Joseph Trotter at the Scripps Research Institute, La Jolla, CA). A minimum of 500,000 events were collected. FACS analysis of each probe was performed in triplicate. The frequency of cEPCs in peripheral blood was determined by a two-dimensional side-scatter/fluorescence dot-plot analysis of the samples after exclusion of annexin V–positive cells and appropriate gating. The exclusion of annexin V–positive cells was performed to exclude contamination with apoptotic cells in our positive population. EPC counts are expressed as a percentage of total PBMCs in each patient or control subject.

### Enzyme-linked Immunosorbent Assay

Serum concentration of VEGF and angiopoietin-2 (Ang-2) was assessed with an enzyme-linked immunosorbent assay kit (R&D Systems) in triplicate samples obtained from 5 ml of serum. Enzyme-linked immunosorbent assay was performed according to the manufacturer’s instructions.

### Statistical Analysis

Data analyses were performed by SPSS software, version 20.0 (SPSS, Chicago, IL). For inner-group comparison at different time points and intergroup comparison, the Kruskal–Wallis test was followed by post hoc testing (Kolmogorov–Smirnov test). The Mann–Whitney *U*-test was used for pairwise comparisons.

*P* < 0.05 was considered to be statistically significant. Quantitative data are presented as mean ± standard deviation.

## Results

### Patient Population

Eleven patients with G3 soft tissue sarcoma (3 men), 11 patients with in-transit metastasized melanoma (3 men), and 15 healthy volunteers were enrolled onto this study. Patient characteristics are listed in Tables 1 and 2.

**Table 2:** cEPC, VEGF, and Ang-2 levels before treatment

Group	*n*	Mean ± SE of:		
		cEPC (% of PBMC)	VEGF (pg/ml)	Ang-2 (pg/ml)
Sarcoma	11	0.179 ± 0.190	359 ± 157	2929 ± 960
Malignant melanoma	11	0.110 ± 0.073	310 ± 303	2564 ± 665
Healthy controls	15	0.025 ± 0.018**	27 ± 13*	1665 ± 445***

*cEPC* circulating endothelial progenitor cell, *VEGF* vascular endothelial growth factor, *Ang*-*2* angiopoietin-2, *PBMC* peripheral blood mononuclear cell

* *P* < 0.001, ** *P* < 0.01, *** *P* < 0.05 vs. sarcoma and malignant melanoma

There was no statistical difference in mean age between the ILP with rhTNF-α and melphalan (55 ± 20 years), the ILP with chemotherapy alone (59 ± 17 years), and the healthy volunteers (42 ± 13 years).

### Circulating Endothelial Progenitor Cells (cEPC) before Treatment

**Fig. 1:**
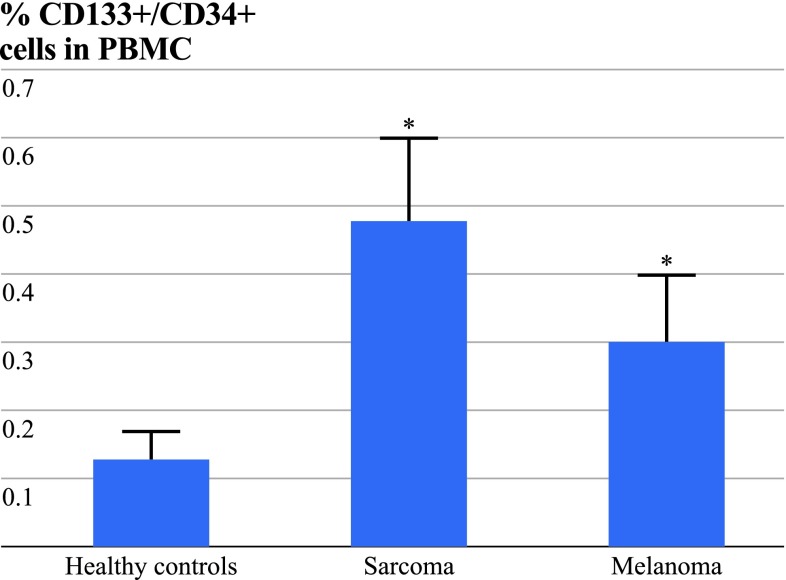
CD133- and CD34-positive cells in PBMC in healthy controls compared to sarcoma and melanoma patients before treatment. CD133- and CD34-positive cells in PBMC are significantly increased in sarcoma and in-transit metastasized melanoma patients compared to healthy controls (*P < 0.01 vs. healthy controls). Data are displayed as mean ± SD; *P* < 0.05 was considered to be statistically significant

The percentage of the hematopoietic stem cells, defined as positive staining for CD34 and CD133, was significantly increased in in-transit metastasized melanoma (0.30 ± 0.10 %) and soft tissue sarcoma (0.48 ± 0.12 %) patients compared to the healthy controls (0.13 ± 0.04 %; *P* < 0.001 each, Fig. 1). The percentage of VEGFR-2^+^ cells within the population of CD34^+^/CD133^+^ cells was measured, thereby defining cEPCs in our study. These findings correspond to cEPC in sarcoma patients of 0.179 ± 0.190 % and in melanoma patients of 0.110 ± 0.073 % versus healthy controls of 0.025 ± 0.018 % (Table 2, Fig. 1; *P* < 0.01 each).

### VEGF and Ang-2 Levels before Treatment

We found significantly increased mean VEGF levels in both sarcoma and melanoma patients (sarcoma 359 ± 157 pg/ml, melanoma 310 ± 303 pg/ml) in comparison to healthy controls (27 ± 13 pg/ml; *P* = 0.001 each). No differences between the treatment groups and the tumor type were observed before treatment (Table 2).

Ang-2 levels before treatment were significantly lower in healthy controls (1665 ± 445 pg/ml) compared to sarcoma (2929 ± 960 pg/ml, *P* = 0.033) and melanoma (2564 ± 665 pg/ml, *P* = 0.045) patients. In regard to VEGF and Ang-2 levels, no differences between the two treatment groups and between the tumor types were observed before treatment (Table 2).

### Effect of ILP with rhTNF-α versus Chemotherapy Alone on cEPC

**Fig. 2:**
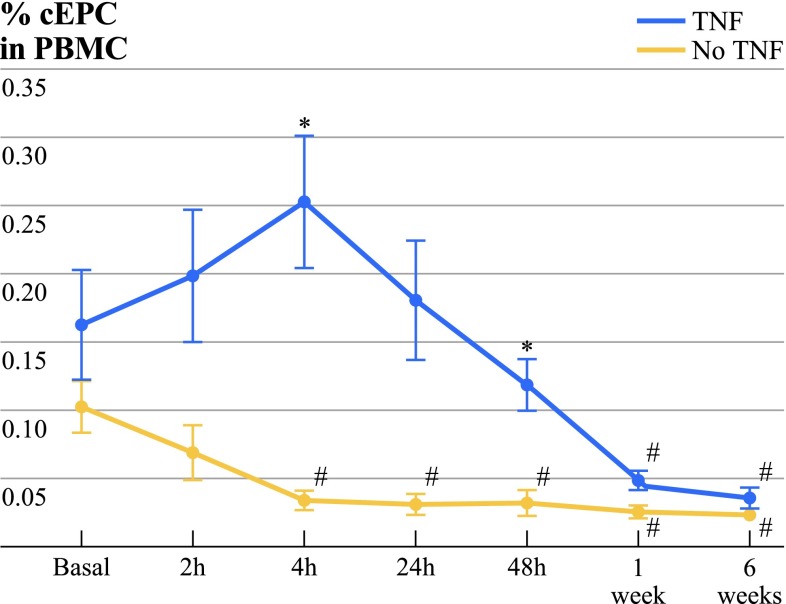
cEPC before and after ILP with or without rhTNF-α. cEPC did not differ significantly before ILP (basal) between the investigated groups. The amount of cEPC was significantly higher 4 and 48 h after ILP with rhTNF-α (TNF) compared to melphalan and cisplatin (no TNF) (*P < 0.05). Compared to basal values, cEPC were significantly lower in no TNF starting at 2 h after ILP and 1 and 6 weeks in TNF after ILP (#*P* < 0.05). Data are displayed as mean ± SEM; *P* < 0.05 was considered to be statistically significant

In ILP with rhTNF-α, cEPC levels were higher compared to ILP with chemotherapy at time points 4 h (TNF 0.252 ± 0.206 %; no TNF 0.034 ± 0.018 %; *P* = 0.037), 24 h (TNF 0.180 ± 0.186 %; no TNF 0.031 ± 0.019 %; *P* = 0.069), 48 h (TNF 0.118 ± 0.081 %; no TNF 0.032 ± 0.022 %; *P* = 0.037), and 1 week (TNF 0.048 ± 0.023 %; no TNF 0.026 ± 0.011 %; *P* = 0.023, Table 3, Fig. 2).

Comparison between pre- and posttreatment cEPC levels showed significant changes in cEPC numbers. After ILP with chemotherapy alone, cEPC numbers decreased significantly from pretreatment values (basal 0.102 ± 0.046 %) more than 50 % within the first 4 h (4 h, 0.034 ± 0.018 %; *P* = 0.031) until the end of the observation period (6 weeks, 0.023 ± 0.011 %; Table 3, Fig. 2; *P* = 0.001).

The changes in cEPC numbers after ILP with rhTNF-α were followed by an overall normalization after 1 week. Six weeks after ILP, cEPC in rhTNF-α were significantly decreased compared to pretreatment values (*P* = 0.005). After 6 weeks, no difference between the two treatment groups was observed (Fig. 2).

### Effect of ILP with rhTNF-α versus Chemotherapy Alone on VEGF and Ang-2

**Fig. 3:**
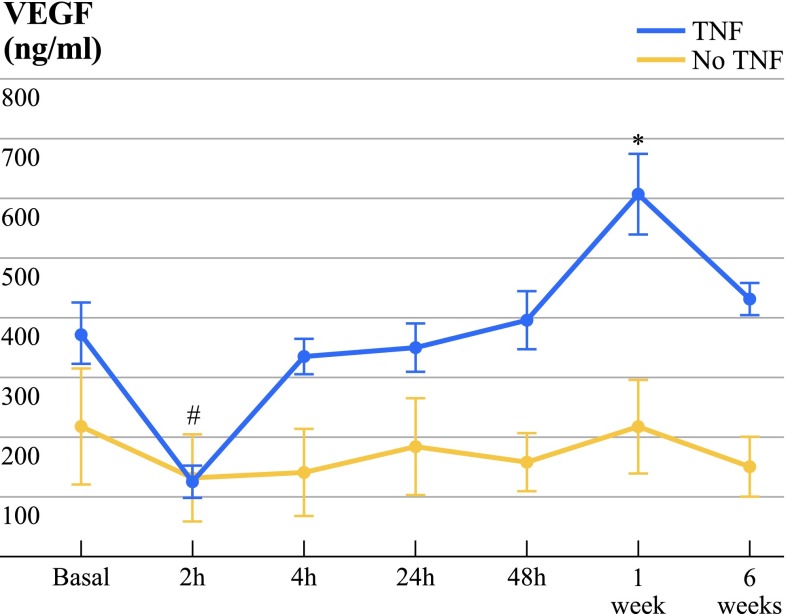
VEGF in patient serum before and after ILP with TNF or without TNF (no TNF). VEGF in serum did not show significant differences between the investigated groups before and after ILP. In rhTNF-α (TNF)-treated patients, VEGF serum levels decreased significantly at 2 h after ILP compared to pretreatment values (basal; #*P* = 0.036). One week after ILP, a significant increase was observed compared to basal values in the TNF group (*P = 0.011). Data are displayed as mean ± SEM; *P* < 0.05 was considered to be statistically significant

In ILP with rhTNF-α, VEGF serum levels were significantly decreased 2 h after ILP (123 ± 119 pg/ml; *P* = 0.036) and significantly elevated 1 week after ILP (606 ± 280 pg/ml; *P* = 0.011) compared to values before ILP treatment (375 ± 231 pg/ml, Table 3, Fig. 3). VEGF serum levels were elevated in ILP with rhTNF-α, although without a statistically significant difference, compared to patients after ILP without rhTNF-α (Table 3, Fig. 3).

**Fig. 4:**
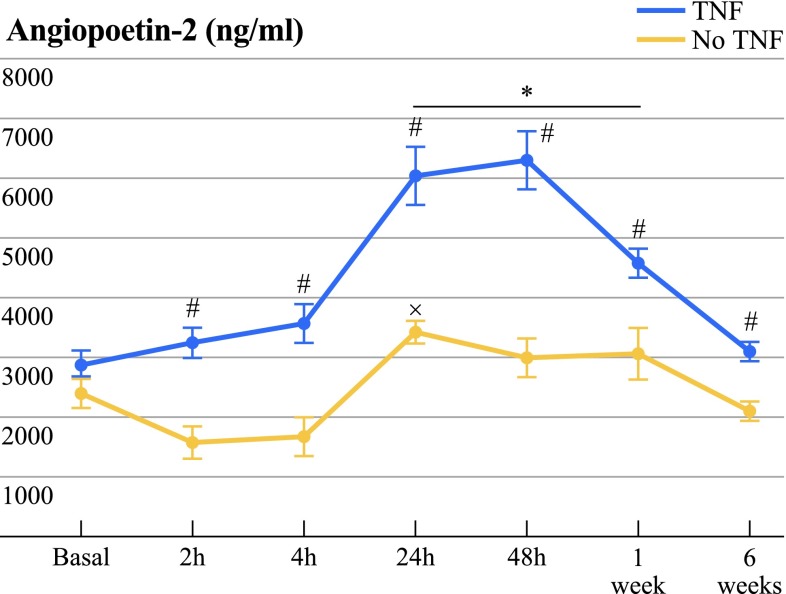
Ang-2 serum levels in patients before and after ILP with TNF or without TNF (no TNF). No significant differences in Ang-2 levels were observed before treatment within the tumor types melanoma and sarcoma and the treatment groups of ILP with rhTNF-α (TNF) compared to cisplatin and melphalan alone (no TNF). After ILP, Ang-2 differed significantly at all points of measurement between the two treatment groups, TNF and no TNF (#*P* < 0.021). A significant increase compared to basal values was found 24 h, 48 h, and 1 week after ILP in the TNF group (*P < 0.001). In no TNF patients were Ang-2 levels significantly higher 24 h after ILP compared to basal values (×*P* < 0.05). Data are displayed as mean ± SEM; *P* < 0.05 was considered to be statistically significant

The course of Ang-2 levels in ILP with rhTNF-α differed significantly compared to ILP with cisplatin/melphalan from the time point 2 h (TNF 3248 ± 1009 pg/ml; no TNF 1585 ± 665 pg/ml, *P* = 0.003) until the end of the observation period after 6 weeks (TNF 3094 ± 469 pg/ml; no TNF 2197 ± 415 pg/ml; *P* = 0.002, Table 3, Fig. 4). In rhTNF-α-treated patients, a significant peak of Ang-2 was observed at 24 h (6024 ± 2085 pg/ml, *P* = 0.000), 48 h (6286 ± 2117 pg/ml, *P* = 0.001), and 1 week (4555 ± 762 pg/ml, *P* = 0.001) compared to values before ILP (2929 ± 960 pg/ml). Within the no-TNF group, a significant increase of Ang-2 was observed 24 h after ILP (3404 ± 417 pg/ml, *P* = 0.031) compared to basal values (2402 ± 606 pg/ml, Table 3, Fig. 4).

After treatment, no significant differences were observed between the tumor types studied.

## Discussion

The study of antiangiogenic strategies are progressing quickly, with the aim of limiting and/or inhibiting tumor progression. Until now, no marker exists to monitor the effect of antiangiogenic treatment as an anticancer therapy.^19^

We describe what is to our knowledge the first quantitative analysis of subsets of circulating VEGFR-2^+^ BMD progenitor cells in the peripheral blood of sarcoma and melanoma patients undergoing ILP. This study demonstrates an increased mobilization of cEPCs in both sarcoma and melanoma patients compared to healthy individuals. We found that levels of cEPC defined by CD133, CD34, and VEGFR-2 were significantly higher during early reperfusion in ILP with TNF-α compared to ILP with chemotherapy alone. In parallel, serum concentrations of Ang-2 were found to undergo significant changes after ILP. A significant increase of Ang-2 in ILP with rhTNF-α was observed 24 and 48 h after ILP compared to pretreatment values in that group (Fig. 4). This means that ILP with rhTNF-α in combination with melphalan might mobilize VEGFR-2-positive progenitors from the bone marrow, followed by Ang-2 expression of EPCs or endothelium. A big part of the explanation must be the consequence of rhTNF-α treatment, causing a higher degree of ischemia and anoxia in the perfused limb. Reperfusion results in a higher risk for severe ischemia–reperfusion injury.^20^ Increased Ang-2 has been reported in response to ischemia–reperfusion and hypoxia.^20^,^21^ Increased Ang-2 has also been shown to be associated with increased endothelial apoptosis.

Growing evidence suggests that BMD EPCs circulate in the blood and play an important role in the formation of new blood vessels.^22^,^23^ Moreover, it is now established that tumor vasculature is not necessarily only derived from endothelial cell sprouting; instead, cancer tissue can acquire its vasculature by alternative mechanisms.^24^ Studies in animals show that EPCs participate in tumor angiogenesis, thereby enhancing tumor growth.^25^ Mobilization of cEPCs from the bone marrow critically depends on the activation of metalloproteinases and up-regulation of adhesion molecules. This is most likely mediated by soluble factors such as VEGF. EPCs provide both instructive (release of proangiogenic cytokines) and structural (vessel incorporation and stabilization) functions that contribute to the initiation of neoangiogenesis.^26^,^27^ However, the lack of a consensual definition of EPC complicates the interpretation of work in this field.

We have used standard flow cytometry to detect cEPCs, although different approaches have been applied in a variety of patient populations.^14^,^28^–^31^ Among these, flow cytometry and colony-forming assays are the most used methods for quantifying cEPCs. Both of these techniques, however, have serious limitations. Although endothelial cell colony-forming units (CFUs) are widely accepted as an estimate of EPC number and function in cell culture, some important limitations may restrict the assumption that endothelial cell CFUs accurately reflect EPC numbers. Shantsila et al., comparing CFU units to flow cytometry, noted that endothelial cell CFU counts represent the cumulative characteristics of EPC quantity and their functional characteristics, and cannot be reliably used for the estimation of EPC numbers in peripheral blood or bone marrow.^32^ They conclude by suggesting that flow cytometry may be the best technique for EPC quantification. Although the exact phenotype of cEPCs is still controversially discussed, the presence of CD34, CD133, and VEGFR-2 seems to be well supported and is therefore used in this study.^29^,^33^

Recent evidence, however, calls into question the ability of BMD EPC to act as a bona fide precursor for adult vasculogenesis.^34^ Wickersheim et al. demonstrated in a murine model that local VEGF production induces a massive infiltration of BMD cells in tumors but does not lead to vessel wall integration of these cells, suggesting that during tumor progression, vascularization occurs primarily via classical tumor angiogenesis.^35^ In contrast, several groups reported significant incorporation of bone marrow EPCs into the vessel wall of tumors.^15^,^36^ In brief, cEPC could be part of both tumor vascularization, inducing paracrine effects on the growth and progression of tumors. Tissue hypoxia present in tumors is considered to be central to this paracrine mechanism. VEGF expression is increased locally within the hypoxic tissue itself, and that in turn stimulates the recruitment of progenitor cells to the hypoxic site.^23^,^37^ Many factors are described to play important roles in mobilizing EPCs.^22^,^38^ Among them are growth factors, such as VEGF, placental growth factor, erythropoietin, and Ang-2; proinflammatory cytokines such as granulocyte macrophage colony-stimulating factor and granulocyte-colony stimulating factor; chemokines such as stromal cell-derived factor 1; hormones such as estrogens; lipid-lowering and antidiabetic drugs; and physical activity. It has been shown that the serum concentration of VEGF correlates with the concentration of EPCs in cancer tissue.^38^ We recently observed a correlation of serum VEGF and cEPC in lung cancer patients.^14^ Fürstenberger et al. observed in patients with primary breast cancer that Ang-2 and VEGF were concomitantly increased with cEPC, suggesting a mobilization by neoadjuvant chemotherapy.^39^

The mean contribution of EPCs to human tumor vasculature in transplantation studies ranged from 1 % to 12 %.^40^ Purhonen et al. reported in animal models that BMD or other endothelial precursors did not contribute to tumor vasculature at all.^41^ As proof that the significant intraluminal incorporation of EPC into tumor vasculature is challenging, the biological role of EPC in tumor angiogenesis was often questioned. Several reports demonstrate, however, that the specific ablation of BMD EPC results in significant impaired tumor growth and vascularization.^36^,^42^ It is a matter of discussion that even with low vessel incorporation, the paracrine effects of EPC may be critical for tumor angiogenesis. Therefore, the differences in EPC incorporation in previously published reports may not only be due to the diversity of tumor models and types studied, but also to the temporal differences in tumor development at the time of study.^23^

The induction of cEPC after ILP with TNF-α and melphalan can be specifically attributed to rhTNF-α, as we have demonstrated that ILP with cytostatic drugs alone induced a significant decrease in cEPC starting 4 h after ILP. After a period of 6 weeks after ILP, cEPC were significantly lower in both investigated groups than before treatment and did not differ from values of healthy volunteers. In our series, significant differences were observed between the treatment regimen of ILP. No differences in principle between the different tumor types have been observed. Although VEGF levels tended to be higher in rhTNF-α-treated patients, no significant difference was observed. However, cEPC and Ang-2 levels were significantly higher at different points of measurements in rhTNF-α patients.

From the observations in our study, we conclude that increased amounts of cEPCs are recruited in sarcoma and in-transit metastasized melanoma patients, most likely for vasculogenesis and paracrine effects, which stimulate angiogenic activity of resting mature endothelial cells. Moreover, our study suggests an association of cEPC numbers with the applied therapeutic agents via ILP.

EPCs may exert an important function as an endogenous stimulus of tumor vasculogenesis. Further studies have to be conducted to reveal whether EPC counts could be a useful tool to decide whether to continue current treatment, thus limiting the use of inefficient and costly therapies.

## Electronic supplementary material

Below is the link to the electronic supplementary material.

Supplementary material 1 (DOCX 127 kb)

## References

[1] Eggermont AM, Schraffordt Koops H, Lienard D (1996). Isolated limb perfusion with high-dose tumor necrosis factor-alpha in combination with interferon-gamma and melphalan for nonresectable extremity soft tissue sarcomas: a multicenter trial. J Clin Oncol..

[2] Hohenberger P, Latz E, Kettelhack C, Rezaei AH, Schumann R, Schlag PM (2003). Pentoxifyllin attenuates the systemic inflammatory response induced during isolated limb perfusion with recombinant human tumor necrosis factor-alpha and melphalan. Ann Surg Oncol..

[3] Tunn PU, Kettelhack C, Durr HR (2009). Standardized approach to the treatment of adult soft tissue sarcoma of the extremities. Recent Results Cancer Res..

[4] Kettelhack C, Hohenberger P, Schulze G, Kilpert B, Schlag PM (2000). Induction of systemic serum procalcitonin and cardiocirculatory reactions after isolated limb perfusion with recombinant human tumor necrosis factor-alpha and melphalan. Crit Care Med..

[5] Utikal J, Zimpfer A, Thoelke A (2006). Complete remission of multiple satellite and in-transit melanoma metastases after sequential treatment with isolated limb perfusion and topical imiquimod. Br J Dermatol..

[6] Balkwill FR (1992). Tumor necrosis factor and cancer. Prog Growth Factor Res..

[7] Renard N, Lienard D, Lespagnard L (1994). Early endothelium activation and polymorphonuclear cell invasion precede specific necrosis of human melanoma and sarcoma treated by intravascular high-dose tumor necrosis factor alphy (rTNF alpha). Int J Cancer..

[8] Davidoff AM, Ng CY, Brown P (2001). Bone marrow–derived cells contribute to tumor neovasculature and, when modified to express an angiogenesis inhibitor, can restrict tumor growth in mice. Clin Cancer Res..

[9] Asahara T, Masuda H, Takahashi T (1999). Bone marrow origin of endothelial progenitor cells responsible for postnatal vasculogenesis in physiological and pathological neovascularization. Circ Res..

[10] Gao D, Nolan DJ, Mellick AS, Bambino K, McDonnell K, Mittal V (2008). Endothelial progenitor cells control the angiogenic switch in mouse lung metastasis. Science..

[11] Kaplan RN, Riba RD, Zacharoulis S (2005). VEGFR1-positive haematopoietic bone marrow progenitors initiate the pre-metastatic niche. Nature..

[12] Rafii S, Lyden D (2008). Cancer. A few to flip the angiogenic switch. Science..

[13] Taylor M, Rossler J, Geoerger B (2009). High levels of circulating VEGFR2^+^ bone marrow–derived progenitor cells correlate with metastatic disease in patients with pediatric solid malignancies. Clin Cancer Res..

[14] Nowak K, Rafat N, Belle S (2010). Circulating endothelial progenitor cells are increased in human lung cancer and correlate with stage of disease. Eur J Cardiothorac Surg..

[15] Shaked Y, Ciarrocchi A, Franco M (2006). Therapy-induced acute recruitment of circulating endothelial progenitor cells to tumors. Science..

[16] Wieberdink J, Benckhuysen C, Braat RP, van Slooten EA, Olthuis GA (1982). Dosimetry in isolation perfusion of the limbs by assessment of perfused tissue volume and grading of toxic tissue reactions. Eur J Cancer Clin Oncol..

[17] Kettelhack C, Hohenberger P, Schulze G, Kilpert B, Schlag PM (2000). Induction of systemic serum procalcitonin and cardiocirculatory reactions after isolated limb perfusion with recombinant human tumor necrosis factor-alpha and melphalan. Crit Care Med..

[18] Rafat N, Hanusch C, Brinkkoetter PT (2007). Increased circulating endotheliel progenitor cells in septic patients: correlation with survival. Crit Care Med..

[19] Bhatt RS, Seth P, Sukhatme VP (2007). Biomarkers for monitoring antiangiogenetic therapy. Clin Cancer Res..

[20] Ray PS, Estrada-Hernandez T, Sasaki H, Zhu L, Maulik N (2000). Early effects of hypoxia/reoxygenation on VEGF, Ang-1, Ang-2 and their receptors in the rat myocardium: implications for myocardial angiogenesis. Mol Cell Biochem..

[21] Tuo QH, Zeng H, Stinnett A (2008). Critical role of angiopoietins/Tie-2 in hyperglycemic exacerbation of myocardial infarction and impaired angiogenesis. Am J Physiol Heart Circ Physiol..

[22] Asahara T, Kawamoto A (2004). Endothelial progenitor cells for postnatal vasculogenesis. Am J Physiol..

[23] Janic B, Arbab AS (2010). The role and therapeutic potential of endothelial progenitor cells in tumor neovascularization. ScientificWorldJournal..

[24] Dome B, Hendrix MC, Paku S, Tovari J, Timar J (2007). Alternative vascularization mechanisms in cancer. Am J Pathol..

[25] Lyden D, Hattori K, Dias S (2001). Impaired recruitment of bone-marrow-derived endothelial and hematopoietic precursor cells blocks tumor angiogenesis and growth. Nat Med..

[26] Dome B, Timar J, Dobos J (2006). Identification and clinical significance of circulating endothelial progenitor cells in human non-small cell lung cancer. Cancer Res..

[27] Yoon CH, Hur J, Park KW (2005). Synergistic neovascularization by mixed transplantation of early endothelial progenitor cells and late outgrowth endothelial cells: the role of angiogenic cytokines and matrix metalloproteinases. Circulation..

[28] Khan SS, Solomon MA, McCoy JP (2005). Detection of circulating endothelial cells and endothelial progenitor cells by flow cytometry. Cytometry B Clin Cytom..

[29] Peichev M, Neiyer A, Pereira D (2000). Expression of VEGFR-2 and AC133 by circulating human CD34(+) cells identifies a population of functional endothelial precursors. Blood..

[30] Hill JM, Zalos G, Halcox JP (2003). Circulating endothelial progenitor cells, vascular function, and cardiovascular risk. N Engl J Med..

[31] Rafat N, Beck G, Schulte J, Tuettenberg J, Vajkoczy P (2010). Circulating endothelial progenitor cells in malignant gliomas. J Neurosurg..

[32] Shantsila E, Watson T, Tse HF, Lip GY (2007). Endothelial colony forming units: are they a reliable marker of endothelial progenitor cell numbers?. Ann Med..

[33] Urbich C, Dimmeler S (2004). Endothelial progenitor cells: characterization and role in vascular biology. Circ Res..

[34] Patenaude A, Parker J, Karsan A (2010). Involvement of endothelial progenitor cells in tumor vascularization. Microvasc Res..

[35] Wickersheim A, Kerber M, de Miguel LS, Plate KH, Machein MR (2009). Endothelial progenitor cells do not contribute to tumor endothelium in primary and metastatic tumors. Int J Cancer..

[36] Nolan DJ, Ciarrocchi A, Mellick AS (2007). Bone marrow–derived endothelial progenitor cells are a major determinant of nascent tumor neovascularization. Genes Dev..

[37] Grunewald M, Avraham I, Dor Y (2006). VEGF-induced adult neovascularization: recruitment, retention, and role of accessory cells. Cell..

[38] Spring H, Schüler T, Arnold B, Hämmerling GJ, Ganss R (2005). Chemokine direct endothelial progenitors into tumor neovessels. Proc Natl Acad Sci U S A..

[39] Fürstenberger G, von Moos R, Lucas R (2006). Circulating endothelial cells and angiogenic serum factors during neoadjuvant chemotherapy of primary breast cancer. Br J Cancer..

[40] Peters BA, Diaz LA, Polyak K (2005). Contribution of bone marrow–derived endothelial cells to human tumor vasculature. Nat Med..

[41] Purhonen S, Palm J, Rossi D (2008). Bone marrow–derived circulating endothelial precursors do not contribute to vascular endothelium and are not needed for tumor growth. Proc Natl Acad Sci U S A..

[42] Singh K, Mogare D, Giridharagopalan RO, Gogiraju R, Pande G, Chattopadhyay S (2007). p53 target gene SMAR1 is dysregulated in breast cancer: its role in cancer cell migration and invasion. PLoS One..

