# Supplementary material for: Alterations of Circulating Bone Marrow–Derived VEGFR-2+ Progenitor Cells in Isolated Limb Perfusion With or Without rhTNF-α
Source: Ann Surg Oncol. 2012 Sep 5;20(11):3694–701. doi: 10.1245/s10434-012-2637-3 (PMC3764318; doi:10.1245/s10434-012-2637-3)
Supplement: Supplementary file 1 — Supplementary material 1 (DOCX 127 kb) [file 10434_2012_2637_MOESM1_ESM.docx]

**Table 3:**

Values of cEPC in patient blood, VEGF and Angiopoetin-2 in patient serum before and after ILP with rhTNF-α versus No TNF (chemotherapy with cisplatin and melphalan alone) are displayed. Data are given in mean ± SD.

Levels of significance are depicted as followed: * means p<0.05, ^#^ means p<0.01, ^+^ means p<0.001 versus either No TNF (depicted as ^a^) or to basal values before treatment (depicted as ^b^).

**cEPC** did not differ significantly before ILP (basal) between the investigated groups. The amount of cEPC in rhTNF-α (TNF) was significantly higher at 4hrs, 48 hrs and 1 week compared to No TNF and were significantly lower after a period of 1 and 6 weeks compared to basal values. After ILP with chemotherapy alone, cEPC numbers decreased significantly from basal values within the first 4hrs until the end of the observation period.

**VEGF** serum levels in rhTNF-alpha (TNF) treated patients decreased significantly at 2hrs after ILP compared to pre-treatment values. One week after ILP a significant increase was observed compared to basal values in the TNF group.

**Angiopoetin-2** after ILP did differ at all points of measurement significantly between the two treatment groups TNF and No TNF. A significant increase compared to basal values was found 24h, 48h and 1 week after ILP in the TNF group.

|  | **ILP with** | **Basal** | **2h** | **4h** | **24h** | **48h** | **1 week** | **6 weeks** |
| --- | --- | --- | --- | --- | --- | --- | --- | --- |
|  |  |  |  |  |  |  |  |  |
| **cEPC** | **rhTNF-α** | 0.162 ± 0.102 | 0.198 ± 0.206 | *^a^ 0.252 ± 0.206 | 0.180 ± 0.186 | *^a^ 0.118 ± 0.081 | *^a^ *^b^ 0.048 ± 0.0023 | ^#b^ 0.035 ± 0.026 |
| [% of PBMC] | **No TNF** | 0.102 ± 0.046 | 0.068 ± 0.050 | *^b^ 0.034 ± 0.018 | *^b^ 0.031 ± 0.019 | *^b^ 0.032 ± 0.022 | ^#b^ 0.026 ± 0.0011 | ^+b^ 0.023 ± 0.011 |
|  |  |  |  |  |  |  |  |  |
| **VEGF** | **rhTNF-α** | 376 ± 231 | *^b^ 123 ± 119 | 335 ± 164 | 351 ± 189 | 396 ± 216 | *^b^ 606 ± 280 | 430 ± 169 |
| [pg/ml] | **No TNF** | 219 ± 240 | 133 ± 178 | 140 ± 176 | 184 ± 196 | 158 ± 120 | 217 ± 191 | 151 ± 121 |
|  |  |  |  |  |  |  |  |  |
| **Ang-2** | **rhTNF-α** | 2929 ± 960 | ^#a^ 3248 ± 1009 | ^#a^ 3553 ± 1345 | ^#a^ ^+b^ 6024 ± 2085 | ^#a^ ^+b^ 6286 ± 2117 | ^#a^ ^+b^4555 ± 762 | ^#a^ 3094 ± 469 |
| [pg/ml] | **No TNF** | 2402 ± 606 | 1585 ± 665 | 1673 ± 797 | ^#b^ 3403 ± 417 | 2996 ± 800 | 3087 ± 1207 | 2197 ± 415 |
